# Genetic Testing of Movements Disorders: A Review of Clinical Utility

**DOI:** 10.5334/tohm.835

**Published:** 2024-01-08

**Authors:** Dennis Yeow, Laura I. Rudaks, Sue-Faye Siow, Ryan L. Davis, Kishore R. Kumar

**Affiliations:** 1 Translational Neurogenomics Group, Neurology Department & Molecular Medicine Laboratory, Concord Repatriation General Hospital, Concord, NSW, Australia; 2 Concord Clinical School, Sydney Medical School, Faculty of Health & Medicine, University of Sydney, Concord, NSW, Australia; 3 Rare Disease Program, Garvan Institute of Medical Research, Darlinghurst, NSW, Australia; 4 Department of Neurology, Prince of Wales Hospital, Randwick, NSW, Australia; 5 Neuroscience Research Australia, Randwick, NSW, Australia; 6 Department of Clinical Genetics, Royal North Shore Hospital, St Leonards, NSW, Australia; 7 Neurogenetics Research Group, Kolling Institute, School of Medical Sciences, Faculty of Medicine and Health, University of Sydney and Northern Sydney Local Health District, St Leonards, NSW, Australia; 8 School of Clinical Medicine, University of New South Wales, Sydney, NSW, Australia

**Keywords:** Genetics, Diagnosis, Movement Disorders, Clinical Utility

## Abstract

Currently, pathogenic variants in more than 500 different genes are known to cause various movement disorders. The increasing accessibility and reducing cost of genetic testing has resulted in increasing clinical use of genetic testing for the diagnosis of movement disorders. However, the optimal use case(s) for genetic testing at a patient level remain ill-defined. Here, we review the utility of genetic testing in patients with movement disorders and also highlight current challenges and limitations that need to be considered when making decisions about genetic testing in clinical practice.

**Highlights:**

The utility of genetic testing extends across multiple clinical and non-clinical domains. Here we review different aspects of the utility of genetic testing for movement disorders and the numerous associated challenges and limitations. These factors should be weighed on a case-by-case basis when requesting genetic tests in clinical practice.

## Introduction

The increasing accuracy, accessibility and decreasing cost of diagnostic genetic testing, largely due to advances in and dominance of next generation sequencing (NGS) technologies, has led to the discovery of a myriad of disease-causing genes over the last two decades [1,2,3]. Currently, variants in more than 500 genes are recognized to cause movement disorders and many more gene-disease associations have been reported but require confirmation [4]. While this has led to improved understanding of movement disorder etiology and pathogenesis, defining and quantifying the overall utility of genetic testing for individual patients with movement disorders is complex, and yet vital for guiding the clinical use of genetic testing.

The utility of genetic testing extends across multiple clinical (e.g., diagnostic, prognostic, therapeutic, etc.) and non-clinical domains (e.g., social, emotional, cognitive, etc.) [5,6,7]. Here, we review genetic testing for movement disorders through the lens of these utility domains and also summarize the challenges and limitations associated with genetic testing. Careful consideration of these factors will help to better inform clinical decisions regarding the use of genetic testing in movement disorder patients.

## Diagnostic Utility

### Diagnostic Yield

The simplest and most quantifiable metric of diagnostic utility is diagnostic yield, the proportion of genetic diagnoses established for a given cohort using a particular genetic testing approach. Diagnostic yield is dependent on multiple factors, including but not limited to, the characteristics of the target population (e.g., type of movement disorder, presence/absence of family history, age at symptom onset, ethnic background), the type of genetic testing used (e.g., Sanger sequencing, short-read NGS, long-read sequencing, repeat-expansion testing using Southern blot and/or repeat-primed polymerase chain reaction [RP-PCR]), the sequencing target (e.g., single gene, targeted gene panel, whole exome sequencing [WES] or whole genome sequencing [WGS]), whether singleton, trio and/or family data is available for analysis, the type and extent of bioinformatic analysis and how strictly standardized variant curation criteria are applied to the classification of variants [8].

A systematic review of the diagnostic yield of genetic testing in all reported movement disorder cohorts is beyond the scope of this article. Instead, we have summarized the diagnostic yields of a non-exhaustive selection of recent (2021 onwards) studies that used prospectively and uniformly applied, genetic testing approaches in composite movement disorder cohorts, as well as cohorts of three selected common movement disorders; ataxia, dystonia and Parkinson’s disease (PD). The rationale for selection of only recent studies is the rapidly increasing number of genetic loci associated with movement disorders, which makes comparison of studies conducted even several years apart difficult; earlier studies have been summarized elsewhere [9,10]. Retrospective cohort studies were excluded to minimize the potential influence of selection bias and heterogeneously applied genetic testing algorithms on diagnostic yield.

Despite restriction to recent prospectively tested cohorts, comparison between the studies presented in Table 1 is still limited by the heterogeneous nature of the studies, including differing methods for patient selection (e.g., inclusion/exclusion criteria) and contexts in which the genetic testing was performed (e.g., genetic testing on a clinical basis vs. research basis). Accepting these limitations, the overall diagnostic yield from the studies for composite movement disorder cohorts was 21–32%, giving an overall estimate of the diagnostic yield capacity of current genetic testing technologies for movement disorders. However, given that most of these studies recruited patients from large tertiary referral centers often with highly selected patient cohorts and some studies analyzed genetic testing that was performed on a research basis, it may be difficult to estimate the yield of these genetic testing strategies when applied on a clinical basis to real world movement disorder patients in the general community.

**Table 1:** Diagnostic yield for genetic testing in a non-exhaustive selection of prospectively tested movement disorder cohorts from 2021–2023.


AUTHORS, YEAR, COUNTRY	TEST (NUMBER OF GENES)	COHORT RECRUITMENT CRITERIA	AGE AT ONSET (YEARS)	DIAGNOSTIC YIELD	VUS/GUS YIELD

**Composite Movement Disorder Cohorts**					

Eratne et al. (2021) [117], Australia	ES (N/A)	Patients with movement disorders and age of onset 2–60 years (except if clear family history); exclusion of movement disorders likely due to a specific single gene	Median 40; 1–69	21.9% (21/96) Ataxia: 25.0% (7/28) Dystonia: 8.3% (2/24) HSP: 40% (10/25) PD: 11% (2/19)	11.5% (11/96)

Kwong et al. (2021) [77], Hong Kong SAR, China	ES (1394)	Patients with pediatric-onset (< 18 years) movement disorders; unrevealing imaging and neurometabolic investigations	0–13	32.2% (10/31) Ataxia: 0.0% (0/1) Dystonia: 50.0% (2/4) HSP: 40.0% (2/5) Paroxysmal movement disorder: 0.0% (0/1) Combined movement disorder: 30.0% (6/20)	12.9% (4/31)

**Ataxia**					

Benkirane et al. (2021) [118], France	ES (490)	Patients with ataxia; exclusion of Friedreich’s ataxia, SCA1, 2, 3 & 6 and ataxia with vitamin E deficiency	Mean 19; 1–53	45.9% (168/366)	N/A

Galatolo et al. (2021) [119], Italy	TGP (285)	Patients with ataxia; exclusion of acquired etiologies, Friedreich’s ataxia and SCA1, 2, 3, 6, 7, 8, 12 & 17	N/A	33.2% (125/377)	15.6% (59/377)

Balakrishnan et al. (2022) [120], India	TP-PCR (4–10) ± ES/GS (N/A)	Patients with ataxia; exclusion of acquired etiologies	N/A	49.3% (74/150)	N/A

Radziwonik et al. (2022) [121], Poland	TGP (152)	Patients with ataxia; exclusion of acquired etiologies, SCA1, 2, 3, 6, 7, 8, 17 & 36 and DRPLA	Mean 32.7; 7–70	55.2% (16/29)	44.8% (13/29)

da Graca et al. (2022) [122], Brazil	ES (N/A)	Patients with ataxia; exclusion of acquired etiologies, SCA1, 2, 3, 6, 7 & 10, Friedreich’s ataxia and DRPLA	Mean 25.8; 2–71	35.5% (27/76)	N/A

**Dystonia**					

Wu et al. (2022) [16], Taiwan	TGP (72), MLPA (13)	Patients with dystonia; exclusion of secondary etiologies	Mean 41.6 ± SD 20	12.6% (40/318)	11.9% (38/318)

Ahn et al. (2023) [123], South Korea	ES (N/A)	Patients with young-onset (< 40 years) dystonia; exclusion of secondary etiologies, patients with a confirmed genetic dystonia based on single gene tests (*TOR1A, SGCE, PRRT2, PANK2, GCH1*, DRPLA, SCA1, 2, 3, 6, 7 & 17) and levodopa-responsive patients	Mean 19.6 ± SD 10.9	20.9% (9/43)	N/A

Li et al. (2023) [124], China	ES (591)	Patients with isolated dystonia	Mean 25.7 ± SD 16; 4–66	21.6% (19/88)	13.6% (12/88)

PD*					

Muldmaa et al. (2021) [125], Estonia	TGP (9) ± MLPA (5)	Patients with PD	Mean 65.2 ± SD 10.1; 35–83	9.5% (18/189)	10.6% (20/189)

Hill et al. (2022) [42], USA	GS (49)	Patients with PD; exclusion of patients with dementia	N/A	13.3% (27/203)	13.8% (28/203)

Hua et al. (2022) [20], China	ES (24), MLPA (6)	Patients with early-onset (< 50 years) PD; exclusion of other neurodegenerative disorders, psychiatric disorders and severe physical illnesses	Mean 43.4 ± SD 7.1	9.0% (14/155)	14/2% (22/155)

Kovanda et al. (2022) [126], Slovenia, Croatia & Serbia	ES (35), MLPA (8)	Patients with PD with either early-onset (< 50 years) or history of PD in at least one first-degree relative	Mean 47; 24 – 87	10.1% (15/149)	14.8% (22/149)

Do et al. (2023) [127], Vietnam	TGP (20), MLPA (8)	Patients with early-onset (< 50 years) PD	Mean 43.1 ± SD 6.0	21.7% (18/83)	22.9% (19/83)

Sun et al. (2023) [19], China	TGP (116) or ES (N/A), MLPA (8) ± TP-PCR (10)	Patients with either early-onset (< 50 years) or history of PD in at least one family member	Median 42, IQR 14	26.9% (224/832)	N/A

Tay et al. (2023) [128], Malaysia	TGP (115), MLPA (5)	Patients with early-onset (< 50 years) PD	N/A	21.7% (35/161)	14.9% (24/161)


DRPLA, dentatorubral-pallidoluysian atrophy; ES, exome sequencing; GS, genome sequencing; GUS, gene of uncertain significance; HSP, hereditary spastic paraplegia; IQR, interquartile range; MLPA, multiplex ligation-dependent probe amplification; N/A, not available; SCA, spinocerebellar ataxia; SD, standard deviation; TGP, targeted gene panel; TP-PCR, triplet-primed polymerase chain reaction; VUS, variant of uncertain significance. * Diagnostic yields for PD cohorts include both pathogenic & likely pathogenic disease-causing variants, as well as select moderate- and high-penetrance risk alleles/variants in *GBA1* and *LRRK2*.

Of the three specific movement disorder cohorts considered in Table 1, diagnostic yield was highest for ataxia (33–55%), moderate for dystonia (12–21%) and lowest for PD (9–13% in unselected cohorts but increasing up to 26% in enriched subpopulations consisting of early-onset and familial PD). These differing yields could reflect intrinsic differences in the genetic architecture of these disorders and/or differential contributions of non-genetic factors (e.g., environmental, epigenetic) to these disorders [3,11,12]. For each disorder, various phenotypic factors (e.g. age at onset, presence/absence of family history, isolated vs. complex movement disorder) modulate the diagnostic yield and may be considered to guide selection of patients that are most likely to attain a genetic diagnosis using current genetic testing technologies [13,14,15]. For example, younger age at onset is generally associated with increased diagnostic yield in dystonia [15,16], ataxia [17,18], and PD [19,20].

Regarding different sequencing technologies, WES and WGS both generally outperform targeted gene panels in terms of diagnostic yield due to broader coverage of the genome. Similarly, WGS is expected to return higher diagnostic yields than WES or custom gene panels due to coverage of intronic regions and improved ability to detect structural variants and repeat expansion disorders through various bioinformatic analyses [21,22,23,24]. Although no studies have directly compared the diagnostic yield of WGS and WES in movement disorder cohorts, WGS has shown superior yield in other populations [25,26].

Potential explanations for the large number of patients who remain genetically undiagnosed despite use of WES or WGS include: variants located in as yet undiscovered loci, complex variants that are difficult to resolve using current clinically validated sequencing technologies, variant mosaicism particularly where pathogenic variants are present in disease-related tissues but absent from blood, disorders due to polygenic or non-genetic causes and/or patients who have been phenotypically misclassified. For this population, periodic reanalysis of existing WES or WGS data may reveal new diagnoses that increase the diagnostic yield, without the need for repeat sequencing. These additional diagnoses may be achieved due to a combination of factors, including clarification and/or evolution of the patient phenotype, identification of new gene-disease associations, reclassification of variant pathogenicity and/or advancements in bioinformatic approaches. For example, reanalysis of WES data from a cerebellar ataxia cohort five years after initial analysis, resulted in variant reclassification in 24% of cases and a 7% increase in overall diagnostic yield, from 21% to 28% [27]. Similarly, application of a copy number variant analysis tool to existing WES data from a dystonia cohort increased the diagnostic yield by 1.5% [28].

### Other Facets of Diagnostic Utility

Early use of genetic testing in the diagnostic workup of selected movement disorder patients may reduce time to diagnosis and provide cost savings compared to diagnostic strategies that do not emphasize early genetic testing. For example, targeted gene panel analysis in a cohort of dystonia patients was shown to be cheaper (€1822 per case vs. €2660 per case) and result in shorter time to diagnosis (28 vs. 102 days), when compared to theoretical diagnostic strategies (excluding use of NGS tests) formulated by movement disorder experts who were provided with detailed clinical features of the patients in this cohort [29]. Cost and resource savings from early genetic testing may in part derive from the avoidance of unnecessary and/or invasive investigations. For example, a genetic diagnosis of *GCH1*-related dopa-responsive dystonia could obviate the need for a lumbar puncture to assess cerebrospinal fluid neurotransmitter levels. Healthcare resource savings may also come from earlier diagnosis of, and thus earlier opportunities to treat, comorbidities that may not otherwise be considered in the absence of a specific genetic diagnosis (e.g., regular echocardiography to screen for development of cardiomyopathy in patients with Friedreich’s ataxia).

## Prognostic Utility

Confirmation of a genetic diagnosis can provide patients and clinicians with a more specific prognosis than that related to a general syndromic diagnosis (i.e., PD, dystonia, etc.). This is evident in the context of monogenic PD, where patients with *GBA1-* or *SNCA-*associated PD tend to have shorter survival, while patients with *PRKN-* and *LRRK2*-associated PD tend to have a longer disease course compared to idiopathic PD [30].

In addition to clarifying prognosis at a syndromic level, the location and class of a pathogenic variant within a single gene can further inform prognosis. For example, truncating variants in the *ATM* gene typically result in absent ATM kinase function and tend to cause the ‘classic’ ataxia-telangiectasia phenotype associated with early-onset, severe disease and life expectancy between 10–20 years, whereas missense variants that result in some residual ATM kinase function tend to result in later-onset, milder disease with longer survival (> 30 years) [31]. Another example is the well-known association between the length of short tandem repeat expansions and disease onset, rate of progression and survival for Huntington’s disease and several other repeat expansion-related movement disorders [32]. Furthermore, interruptions and/or non-canonical repeat motifs within these repeat tracts, both of which are not well identified using current standard genetic assays, are known to further modulate the phenotype of these movement disorders [33,34,35]. The use of long-read sequencing, which overcomes many of the limitations of other current genetic technologies, has resulted in increasing identification of these repeat expansion variations and better understanding of their impact on clinical phenotype and prognosis [36,37].

While the focus of this article is on diagnostic genetic testing, it is worth noting that genotyping for common polymorphisms across the genome may also provide prognostic utility. For instance, polygenic scores that integrate the effect of multiple common polymorphisms correlate with motor and cognitive progression in PD [38]. While such polygenic scores are currently not used routinely in clinical practice, they have the potential to provide additional personalized prognostic information for patients with movement disorders [39]. Further investigation into their utility is required to inform translation into clinical practice.

The prognostic information provided by genetic sequencing is valued by movement disorder patients, even if genetic diagnosis does not result in changes to management [40,41,42]. For example, one survey-based study of PD patients found that approximately 80% of respondents indicated they would undergo genetic testing in order to specifically inform them of the rate of disease progression [40].

## Therapeutic Utility

Establishing a genetic diagnosis may guide the medical and surgical treatment of several movement disorders, potentially resulting in symptomatic benefit and, in some cases, modification of disease course. In Table 2 we have provided examples of the therapeutic utility of genetic testing in a number of genetic movement disorders; more comprehensive lists of treatable genetic movement disorders have been published previously [43,44,45,46]. While the overall number of genetic movement disorders with approved disease-specific treatments is currently limited, this number is continuously growing. Furthermore, even in the absence of disease-specific treatments with proven efficacy, establishing a genetic diagnosis may facilitate patient recruitment into clinical trials of upcoming novel therapeutic agents.

**Table 2:** Examples of specific therapeutic implications for common genetic movement disorders.


INTERVENTION	CONDITION (GENES/GENES)	TREATMENT/THERAPEUTIC IMPLICATION

Targeted medication	Aceruloplasminemia (*CP*)	Iron chelation [129], fresh frozen plasma [130]

	*ADCY5*-related dyskinesia (*ADCY5*)	Caffeine [49]

	Aromatic L-amino acid decarboxylase deficiency (*DDC*)	Pyridoxine, dopamine agonists, monoamine oxidase inhibitors [131]

	Benign hereditary chorea (*NKX2-1*)	Levodopa [132]

	Cerebrotendinous xanthomatosis (*CYP27A1*)	Chenodeoxycholic acid [133]

	Dopa-responsive dystonia (*GCH1, TH, SPR, PTS, QDPR*)	Levodopa [134]

	Episodic ataxia type 2 (*CACNA1A*) and spinocerebellar ataxia 27B (*FGF14*)	Acetazolamide, 4-aminopyridine [47,48,135,136]

	Friedreich’s ataxia (*FXN*)	Omaveloxolone [50]

	Hypermanganesemia with dystonia (*SLC30A10, SLC39A14*)	Manganese chelation with EDTA [137]

	Niemann-Pick disease type C (*NPC1, NPC2*)	Miglustat [138]

	Paroxysmal kinesigenic dyskinesia (*PRRT2*)	Sodium channel blockers (e.g., carbamazepine) [139]

	Rapid onset dystonia-parkinsonism (*ATP1A3*)	Flunarizine [140]

	Spinocerebellar ataxia 38 (*ELOVL4*)	Docosahexaenoic acid [141]

	Wilson’s disease (*ATP7B*)	Copper chelation [142], zinc [143]

DBS	Parkinson’s disease with *GBA1* variants	Cognitive worsening after DBS of subthalamic nucleus [53,54]

	Certain genetic dystonias (e.g., *TOR1A, TAF1, SGCE, KMT2B*, etc.)	Good response to DBS of globus pallidus internus [56,57,58]

	Rapid onset dystonia-parkinsonism (*ATP1A3*)	Poor response to DBS [59,60,61]

Dietary modification	Abetalipoproteinemia (*MTTP*)	Low-fat diet and supplementation of fat-soluble vitamins [144]

	Ataxia telangiectasia	Nicotinamide riboside supplementation [145]

	Ataxia with vitamin E deficiency (*TTPA*)	Vitamin E supplementation [64]

	Ataxias with coenzyme Q10 deficiency (*ADCK3, ANO10, APTX*)	Coenzyme Q10 supplementation [46]

	Biotin-thiamine responsive basal ganglia disease (*SLC19A3*)	Biotin and thiamine supplementation [146]

	Cerebral creatine deficiency syndromes (*GAMT, GATM*)	Creatine supplementation [147]

	Cerebral folate transport deficiency (*FOLR1*)	Folinic acid supplementation [148]

	GLUT1 deficiency syndrome (*SLC2A1*)	Ketogenic diet [65,66]

	Glutaric aciduria type 1 (*GCDH*)	Low-lysine diet and carnitine supplementation [149]

	Refsum’s disease (*PHYH, PEX7*)	Dietary restriction of phytanic acid [150]

Gene therapy	Aromatic L-amino acid decarboxylase deficiency (*DDC*)	Eladocagene exuparvovec [68]

Trigger avoidance	Alternating hemiplegia of childhood (*ATP1A3*)	Avoid emotional stress, excess physical exertion, water exposure, and temperature extremes [140]

	Biotin-thiamine responsive basal ganglia disease (*SLC19A3*)	Avoid fever [146]

	Episodic ataxia type 2 (*CACNA1A*)	Avoid alcohol, excess physical exertion, emotional stress, and caffeine [135]

	Glutaric aciduria type 1 (*GCDH*)	Avoid catabolic states (e.g., ensure high caloric intake during intercurrent illnesses) [149]

	*POLG*-related mitochondrial disorders (*POLG*)	Avoid valproic acid [69]

	Rapid onset dystonia-parkinsonism (*ATP1A3*)	Avoid alcohol, excess physical exertion, fever, and emotional stress [44,70]


DBS, deep brain stimulation.

### Targeted Medication

While medical management of movement disorders is often based on a syndromic diagnosis, a genetic diagnosis can facilitate a precision medicine approach, with use of specific medications or dosing regimens for individual genetic conditions. Recent examples of movement disorders with specific symptomatic treatments include the recently described spinocerebellar ataxia (SCA) 27B, where the use of 4-aminopyridine has been shown to reduce the severity and frequency of certain symptoms (e.g. diplopia, dizziness, and dysarthria) in up to 86% of patients [47,48], and *ADCY5*-related dyskinesia, where the use of caffeine reduces paroxysmal hyperkinetic movements in up to 87% of patients, likely through the inhibition of adenosine A2A receptor activation of ADCY5 in the striatum [49]. In addition to symptomatic treatments, the number of disease-modifying therapies also continues to grow, e.g., omaveloxolone, an oral small molecule antioxidant that has recently been shown to slow progression and even improve neurological function in patients with Friedreich’s ataxia [50].

An increasing number of novel or repurposed therapeutic agents are in clinical trials for treatment of numerous different genetic movement disorders. Some examples of agents in phase III trials for movement disorders include BIIB122, an oral small molecule LRRK2 inhibitor in patients with *LRRK2*-related PD (NCT05418673) and metformin, which is being repurposed for trial use in patients with Huntington’s disease (NCT04826692).

Lastly, pharmacogenomic testing (genotyping of polymorphisms in genes that relate to drug response and metabolism) may be helpful in predicting the effect of medications in patients with movement disorders. As an example, testing for polymorphisms in genes encoding dopamine receptors and enzymes that metabolize levodopa could potentially be used to help predict efficacy of and likelihood of side effects to levodopa in individual patients with PD [51].

### Deep Brain Stimulation

Although deep brain stimulation (DBS) has well-established efficacy in a variety of movement disorders, there is increasing evidence to show that response to DBS may vary depending on genetic factors. For example, although many monogenic forms of PD generally show good response to DBS [52], several case series of patients with *GBA1* variants have shown a tendency towards more rapid cognitive impairment after DBS of the subthalamic nucleus (STN) [53,54]. However, large prospective studies of DBS in patients with *GBA1* variants stratified by variant type and DBS target (STN vs. globus pallidus interna [GPi]) are required to produce more definitive conclusions. Until such time, given that *GBA1* patients experience favorable and durable motor response to DBS [55], genetic results should not be used as a determinant of DBS eligibility in monogenic PD. Instead, genetic status should be discussed with patients alongside other clinical factors that may impact response to and safety of DBS (e.g., age, comorbidities) to better inform discussions on likely outcomes.

Good motor outcomes from DBS of the GPi have been reported for multiple forms of monogenic dystonia including those related to variants in *TOR1A, TAF1, SGCE*, and *KMT2B* amongst others [56,57,58]. In contrast, DBS of GPi has been found to be generally ineffective in patients with *ATP1A3*-related dystonia-parkinsonism [59,60,61]. However, a few cases involving either a novel *ATP1A3* variant or novel DBS target have shown reasonable response to DBS [62,63] suggesting that, as is the case for PD, gene status alone should not be used to decide on eligibility for DBS.

### Dietary Modification

Several genetic movement disorders respond to specific dietary modifications [44]. In particular, dietary vitamin supplementation may be indicated for genetic deficits in vitamin absorption or utilization e.g., vitamin E supplementation to stabilize and even improve symptoms in *TTPA*-related ataxia with vitamin E deficiency [64]. Conversely, dietary restrictions to reduce accumulation of toxic metabolites or to encourage use of alternative metabolic pathways may also be indicated for some disorders. An example of this is *SLC2A1*-related gluocose-transporter-1 deficiency where the ketogenic diet is used to generate ketone bodies that can provide the central nervous system with an alternative source of energy to glucose, thus preventing the seizures and paroxysmal dyskinesia that normally occur in this disorder [65,66].

### Gene Therapy

Gene therapy aims to alter gene expression or function for therapeutic purposes. Various techniques can be employed, including gene transfer to replace a dysfunctional gene, alteration of gene expression by up- or down-regulation, alteration of mRNA splicing or direct gene editing [67]. Currently, the only approved gene therapy for a genetic movement disorder is eladocagene exuparvovec, an intraputaminally-infused recombinant adeno-associated viral (AAV) vector containing the human *DDC* gene, for treatment of aromatic L-amino acid decarboxylase deficiency, a severe childhood-onset neurometabolic disorder that can present with oculogyric crisis and dystonia. Use of this gene therapy restores dopaminergic synthesis and results in rapid and robust improvements in cognitive and motor function [68].

Gene therapies for many other genetic movement disorders are either in development or clinical trials. Some examples include: LY3884961, an intra-cisterna magna injected AAV-based gene transfer therapy to restore glucocerebrosidase function in PD patients with pathogenic *GBA1* variants (NCT04127578); BIIB094, an intrathecal antisense oligonucleotide designed to knockdown LRRK2 expression in PD patients with gain of function *LRRK2* variants (NCT03976349); and PTC518, an oral small molecule splicing modifier that knocks down *HTT* expression in Huntington’s disease (NCT05358717). Successful development of gene therapies heralds exciting prospects for the treatment of genetic movement disorders.

### Trigger Avoidance

For some movement disorders, a genetic diagnosis may provide guidance regarding medications and/or triggers that should be avoided. For example, although valproic acid is a commonly use medication to treat seizures and occasionally myoclonus, it may precipitate liver failure in and therefore is absolutely contraindicated for patients with *POLG-*related mitochondrial disease, which occasionally presents with various movement disorders including ataxia, parkinsonism, myoclonus, and dystonia [69]. Another example is the acute neurological deterioration seen in *ATP1A3*-related rapid onset dystonia-parkinsonism, which may be precipitated by environmental triggers, including alcohol, excessive exercise, and fever [44,70]. Attempts to avoid relevant triggers should form part of management recommendations for patients with these genetic movement disorders.

### Avoidance/Cessation of Unnecessary Treatment

Establishing a genetic diagnosis may facilitate discontinuation or avoidance of unnecessary treatments targeted at alternative diagnoses. For example, *GCH1*-related dopa-responsive dystonia typically presents with focal lower limb dystonia which, if not diagnosed, has been reported to occasionally be unnecessarily treated with orthopedic surgery aiming to correct the abnormal foot posture [71]. It is also not uncommon to encounter patients with a presumptive diagnosis of an autoimmune neurological disorder who are subsequently diagnosed with a genetic movement disorder, allowing for discontinuation of immunosuppressant medications [72,73]. Although co-occurrence of a genetic movement disorder and an autoimmune neurologic disorder is well described [74,75,76], it remains unclear if these associations are causal (i.e., the genetic disorder predisposes to a secondary autoimmune process) or occur by chance.

### Overall Therapeutic Impact

Despite being a critical component of the overall utility of genetic testing, the therapeutic impacts of establishing a genetic movement disorder diagnosis are reported in only a few diagnostic cohort studies. In a small cohort of pediatric-onset movement disorder patients, 80% (8/10) of genetic diagnoses had specific treatment implications [77] while in another pediatric movement disorder cohort, 38% (10/26) of individuals obtaining a genetic diagnosis received disease-specific treatment [78]. In a larger cohort of dystonia patients, 34.1% (46/135) of the genetic diagnoses were associated with disease-specific treatment (32/46) or screening (14/46) recommendations, although the number of patients in whom such recommendations were enacted and the outcomes of this were not reported [15]. Hence, there is a great need for longitudinal data incorporating objective disease outcome measures to quantify and qualify the long-term therapeutic impact of genetic testing in cohorts of movement disorder patients. This data would help to guide clinical decisions and guidelines surrounding genetic testing in movement disorder patients. Lastly, it should also be noted that the therapeutic impact of genetic testing is not static – it is likely to increase with time as more gene-specific treatments continue to emerge.

## Reproductive Utility

Individuals affected by or at-risk of a genetic movement disorder, and their partners, often face challenges with reproductive decision making [79,80,81]. For patients with a known or suspected genetic movement disorder who are considering family planning, referral to a clinical geneticist and/or genetic counsellor is recommended to facilitate informed reproductive decisions.

A range of reproductive options are available to patients with genetic conditions, including natural conception without genetic testing, prenatal genetic testing *in utero* with the choice to continue or terminate an affected pregnancy, preimplantation genetic testing via *in vitro* fertilization to implant an unaffected embryo, use of unaffected donor sperm or eggs, fostering, adoption, or choosing not to have children [80,81,82]. In addition, the emerging technology of mitochondrial donation offers the ability to reduce the risk of transmitting pathogenic mitochondrial DNA variants, which are the cause of several movement disorders (e.g., m.8993T>G-related neuropathy, ataxia and retinitis pigmentosa syndrome) [83].

Prenatal genetic testing, preimplantation genetic testing, and mitochondrial donation all require a specific genetic diagnosis to have been established, while the other options do not. Surveys of movement disorder patients have demonstrated there is substantial interest in utilization of these reproductive technologies, particularly preimplantation genetic testing. In a group of ten young female presymptomatic Huntington’s disease carriers, all expressed an interest in preimplantation genetic testing to avoid having at-risk children [84]. Similarly, in a survey of 94 patients with SCA, a majority of respondents (61.5%) felt it was important to avoid having an affected child, with preimplantation genetic testing being the most frequently considered reproductive option [82].

## Clinical Utility for Genetic Relatives

The clinical utility of genetic testing should not only be considered from the patient’s perspective but also from the perspective of family members. Establishing a genetic diagnosis in a proband facilitates accurate counselling of family members regarding their risk of inheriting or carrying a genetic variant and provides the opportunity for predictive testing. Predictive testing may provide family members with increased certainty about the future, the ability to make more informed reproductive decisions, and opportunities to enact preventative measures, where available. However, it also has the potential to lead to genetic discrimination by employers and/or insurance providers and increased anxiety, particularly given the lack of curative or disease modifying therapies for many genetic movement disorders. Decisions regarding the use of predictive testing are complex and highly personal; comprehensive pre- and post-genetic test counselling should be mandatory [85] and the “right not to know” of family members must be considered and respected [86].

In addition to the possibility of predictive testing, certain genetic movement disorder diagnoses may even inform clinical care for family members that are asymptomatic carriers. For example, family members of a proband with ataxia-telangiectasia who are heterozygous carriers for pathogenic *ATM* variants will not develop ataxia-telangiectasia themselves, but are at increased risk of developing breast, prostate and pancreatic cancers compared to the general population and thus benefit from early cancer screening to reduce cancer mortality [87].

## Non-Clinical Utility

Although this review focuses on clinical utility, the non-clinical utility of establishing a genetic diagnosis is not insignificant. This includes benefits across social (e.g., increased access to disease-specific social and/or institutional supports), emotional (e.g., increased patient empowerment and sense of agency), cognitive (e.g., the ‘value of knowing’ a diagnosis itself and resolution of a diagnostic odyssey) and knowledge (e.g., advancing scientific understanding of disease mechanisms) domains [88]. These non-clinical facets of utility may be more difficult to quantify than objective clinical measures, such as diagnostic yield or time to diagnosis, but are no less valued by patients and families with genetic disorders. Multiple discrete choice experiment studies performed in these cohorts as well as in members of the general population have demonstrated a high value ascribed to these non-clinical components of genetic testing utility [89,90,91].

## Challenges and Limitations of Genetic Testing of Movement Disorders

While diagnostic genetic testing can provide considerable utility, it is also associated with numerous challenges and limitations which are not trivial [92]. Solutions to these problems have not come easily; many of the challenges of applying high-throughput genetic sequencing in neurology that were present a decade ago [93] still have not been fully resolved.

### Technical & Data Interpretation Issues

Limitations in NGS methodology (e.g., difficulty characterizing GC-rich regions, highly repetitive or homologous regions and complex structural variants) contribute to suboptimal diagnostic yield. Fortunately, improvements in diagnostic yield are being gained through the use of newer genetic technologies (e.g., long-read sequencing, optical genome mapping) that address many of the technical limitations of NGS [1,36,94,95]. Indeed, use of long-read sequencing has contributed to the discovery and/or validation of recently described novel repeat expansion loci as the cause of various movement disorders, including the GAA repeat expansion in *FGF14* that causes SCA 27B [48,96] and TTTCA/TTTTA repeat expansions that cause the familial cortical myoclonic tremor with epilepsy syndromes [97,98]. It is likely there are more movement disorder-causing repeat expansion loci yet to be discovered.

Another issue relating to the use of high throughput genetic sequencing is the increasing identification of variants of uncertain significance (VUS), which are variants for which there is insufficient data to allow for classification as a pathogenic/likely pathogenic or benign/likely benign, and high impact variants in genes of uncertain significance (GUS), where there is no firmly established link between the identified gene and the phenotype of interest. In the era of high-throughput NGS, VUSs and GUSs are common (10.6% to 44.8% of patients in the studies listed in Table 1), occasionally even eclipsing the actual diagnostic rate. VUSs have the potential to be misinterpreted by patients and may elicit negative reactions (e.g., distress over uncertainty, disappointment, frustration) [99]. Reclassification of VUSs and GUSs may occasionally be possible based on further *in silico* analyses or familial segregation studies. However, in most cases, functional studies are required to assess the biologic effect of the specific VUS/GUS and provide sufficient additional evidence for or against pathogenicity. Currently functional studies are often low-throughput and time- and cost-intensive. Furthermore, selection of an appropriate functional assay and model system is often not straightforward [100]. Overall, solutions to this problem require greater global collaboration between clinical and research teams, improvements in bioinformatic analysis pipelines, generation and curation of more comprehensive variant databases, use of more diverse genomic references, and development of reliable, high-throughput functional assays [100,101].

### Logistic Issues

Issues relating to access, cost and funding/insurance coverage for genetic testing hinder the widespread and equitable use of genetic testing. Despite the falling costs of genetic testing with time, recent surveys of movement disorder physicians show that testing costs and limited access to genetic testing and genetic counselling remain major barriers to routine genetic testing of movement disorder patients around the world [102,103,104]. Solutions to this require building genetic and genomics workforce capacity at a local level and also collaborative efforts at a global scale (e.g., the Global Parkinson’s Genetics Program [105]).

With the growing volume of genetic data being generated, logistical issues relating to storage and analysis of large amounts of genomic data as well as data integrity and privacy concerns are becoming increasingly important. Both technologic advances and development of clearer public policy in this area are required to protect the privacy of genetic data moving forward [106].

### Legal, Ethical and Psychological Issues

A potential barrier to more widespread use of genetic sequencing is the risk of genetic discrimination [107]. A survey of patients with PD identified that the impact of genetic testing on the ability to obtain health and life insurance was a common concern [108]. Across the world, different jurisdictions and health systems have enacted regulations at either a legislative or industry level in an attempt to mitigate discrimination by insurance providers based on genetic results [109,110]. However, ongoing genetic discrimination despite these policies [111] indicate a need for continuing review and augmentation of these regulations. In a similar vein, thoughtful public policy is also required to regulate the emerging potential for use of stored genetic data for non-medical (e.g., forensic) purposes [112].

Some patients may experience stigma and/or psychological distress following receipt of a genetic diagnosis. In particular, a systematic review exploring the psychological impact of genetic diagnosis found that unlike most other groups of patients, patients with Huntington’s disease uniquely experienced negative psychological impacts (including depressive symptoms and suicidal ideation) following genetic testing [113]. However, it is possible these symptoms could, at least in part, reflect pre-existing affective disorders or psychiatric manifestations of the disease itself. Provision of adequate pre- and post-test education, genetic counselling and psychological support is required to help mitigate this risk [113].

Other practical issues that benefit from adequate genetic counselling are those relating to incidental and secondary genetic findings [114] and the potential for genetic testing to identify unexpected familial relationships (e.g., non-paternity, consanguinity). Clinical practice relating to management of incidental findings and return of secondary findings varies globally; adherence to clinical practice guidelines on this topic is important to minimize harm [115].

## Genetic Testing for Movement Disorders in Clinical Practice

**Figure 1:**
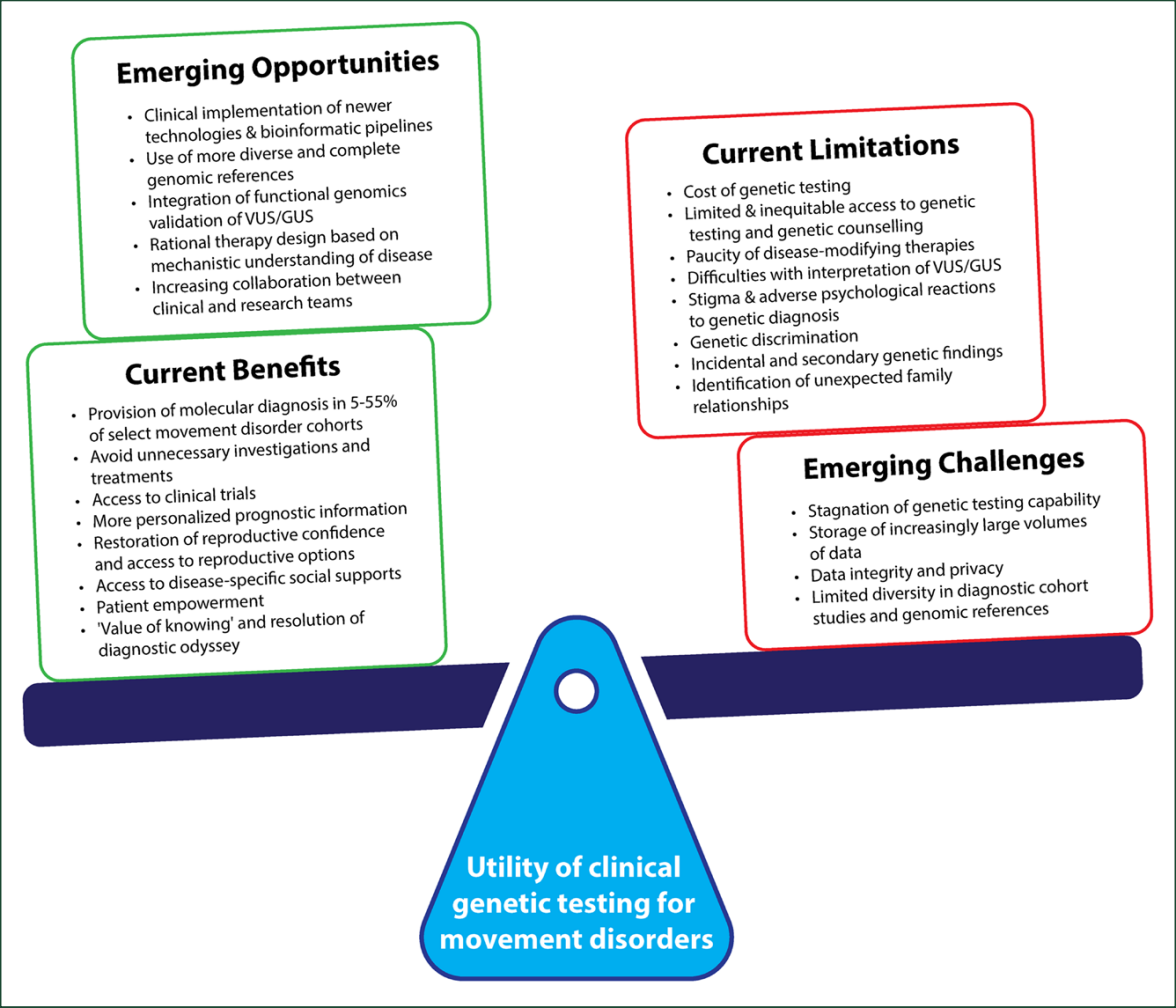
Benefits, challenges & limitations of genetic testing for movement disorders. The balance of benefits, challenges and limitations for genetic testing of movement disorders is dynamic, requiring the routine consideration of both clinical and non-clinical domains. Taking a broad general perspective, the current balance of these ‘pros’ and ‘cons’ likely weighs in favor of genetic testing. However, the exact position of the scales is dependent on individual patient factors, local resource availability and healthcare system contexts. Clinicians must consider these factors holistically when making decisions regarding genetic testing, on a case-by-case basis and in consultation with patients and their families. Emerging opportunities and challenges will continue to shift the balance of these decisions, likely further in favor of genetic testing as improvements in genetic technology and scientific understanding of disease lead to improved diagnosis, prognostication and treatment of genetic movement disorders. GUS, gene of uncertain significance; VUS, variant of uncertain significance.

Although there is a dearth of real-world movement disorder-specific data that systematically weighs the numerous utilities of genetic testing against the aforementioned challenges and limitations, it is our opinion that the current benefits of genetic testing in movement disorders likely outweigh the collective risks, in a general sense (Figure 1). This opinion is shared by half of respondents in a survey of neurologists who were asked about the use of WGS in clinical practice [116]. However, decisions pertaining to genetic testing at an individual patient level in clinical practice (e.g., who to test, at what point in time to test, which test[s] to use) are complex, personal and also dependent on local resources and healthcare system contexts. Therefore, ultimately, these decisions still rely on clinical acumen and case-by-case discussions of the potential benefits and risks with individual patients and their families.

The fast-evolving and dynamic nature of movement disorder genetics means that the balance of benefits and risks shifts continually with time – likely, in our opinion, further in favor of genetic testing, as improvements in genetic technology and scientific understanding of disease lead to increased rates of diagnosis, greater gene- and variant-specific prognostic information and an increasing number of disease-specific therapeutics. Nevertheless, the challenges associated with genetic testing will remain substantial unless there are conscious, collaborative and concerted efforts on the part of all stakeholders towards solving them.

## Conclusion

Diagnostic genetic testing offers considerable utility to patients with movement disorders, and this extends across multiple clinical and non-clinical domains. However genetic testing is also associated with numerous important challenges and limitations that require global collaborative efforts to solve. This review serves as a primer, or refresher, for clinicians looking after movement disorder patients regarding the potential benefits and risks that need to be weighed on a case-by-case basis when making decisions about genetic testing in clinical practice.
